# Peripapillary hyperreflective ovoid mass-like structures: multimodal imaging and associated diseases

**DOI:** 10.3389/fneur.2024.1379801

**Published:** 2024-03-28

**Authors:** Di Xiao, Tsering Lhamo, Yang Meng, Yishuang Xu, Changzheng Chen

**Affiliations:** Department of Ophthalmology, Renmin Hospital of Wuhan University, Wuhan, China

**Keywords:** peripapillary hyperreflective ovoid mass-like structures, axoplasmic stasis, multimodal imaging, optical coherence tomography, optic nerve

## Abstract

Growing evidence has demonstrated that peripapillary hyperreflective ovoid mass-like structures (PHOMS) are novel structures rather than a subtype of optic disc drusen. They correspond to the laterally bulging herniation of optic nerve fibers and are believed to be the marker of axoplasmic stasis. PHOMS present in a broad spectrum of diseases, including optic disc drusen, tilted disc syndrome, papilloedema, multiple sclerosis, non-arteritic anterior ischemic optic neuropathy, optic neuritis, Leber hereditary optic neuropathy, and so on. We focus on the multimodal imaging features, pathophysiological mechanisms of PHOMS, and their association with multiple diseases and healthy people in this review to deepen the ophthalmologists' understanding of PHOMS. Additionally, we provide some new directions for future research.

## 1 Introduction

Peripapillary hyperreflective ovoid mass-like structures (PHOMS) are newly defined imaging structures in the study of optic disc drusen (ODD) (1). Previously, numerous peripapillary lesions identical to PHOMS were recognized as a subtype of ODD (2–4). However, the international organization Optic Disc Drusen Studies (ODDS) Consortium made it clear for the first time in 2018 that this hyperreflective structure was a distinct entity from ODD in a study adopting enhanced depth imaging optical coherence tomography (EDI-OCT) (1). They named the structures PHOMS based on their morphological characteristics on optical coherence tomography (OCT) B-scans (1). PHOMS are believed to correspond to the laterally bulging herniation of the optic nerve fibers and axoplasmic stasis (5, 6). In recent years, PHOMS have been recognized in several neuro-ophthalmic diseases, such as ODD, tilted disc syndrome (TDS), papilloedema, non-arteritic anterior ischemic optic neuropathy (NAION), optic neuritis (ON), Leber hereditary optic neuropathy (LHON) and so on (7–12). Besides, they have also been reported in healthy people (13–15). With the development of multimodal imaging technology, we are able to recognize the morphological characteristics of PHOMS more clearly in the expanding disease spectrum and healthy individuals. In this review, we summarize the multimodal image characteristics and pathophysiologic mechanisms of PHOMS, as well as discuss their association with a variety of ophthalmic disorders and healthy people in order to deepen the clinicians' knowledge of PHOMS. Additionally, we draw attention to a few unresolved issues with these structures and offer potential new directions for research.

## 2 Identification of PHOMS

### 2.1 Optical coherence tomography hallmarks

The emergence of EDI-OCT has greatly improved the acquisition speed and imaging penetration depth, allowing us to observe the fine structure of the laminar cribrosa, the Bruch membrane opening (BMO), and the peripapillary region adjacent to the opening clearly (16). With a clear visualization of the peripapillary structure on EDI-OCT, the ODDS Consortium identified several characteristics of PHOMS that differed from ODD (1). However, their definition of PHOMS was only in the context of ODD, by which a relatively poor consistency in the identification of PHOMS between evaluators was obtained (17). In 2020, Petzold et al. (17) perfected the definition from three aspects, including location, signal, and influence on the adjacent retina based on OCT B-scans, leading to an enhancement in the consistency of PHOMS detection.

**Figure 1:**
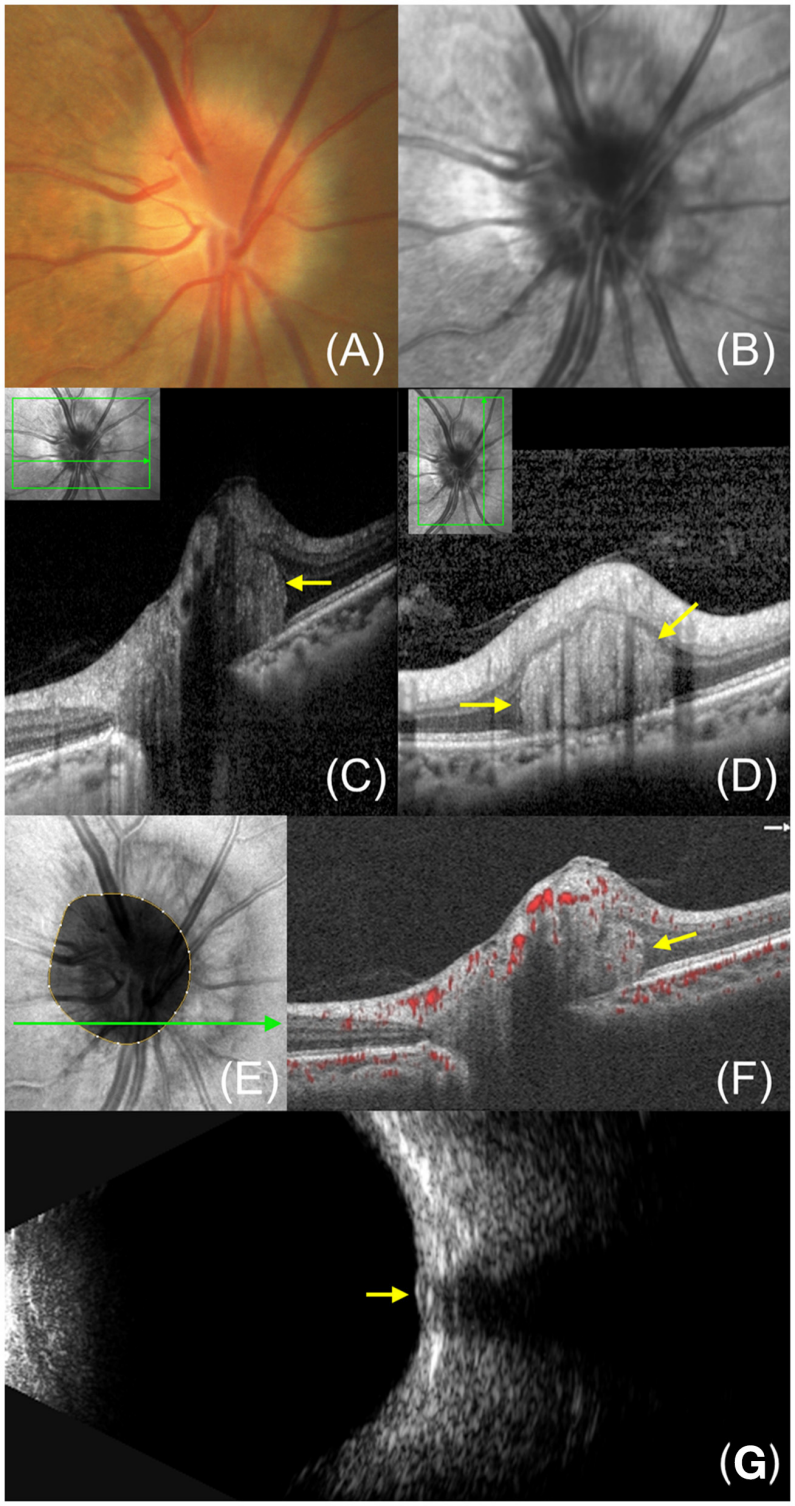
Multimode image features of PHOMS in a 45-year-old female. **(A)** Fundus photography reveals a “C”-shaped halo surrounding the optic disc. **(B)** Infrared reflectance imaging exhibits a clear ring structure corresponding to the blurred edges of the optic disc. **(C, D)** Enhanced depth imaging-OCT image shows PHOMS (yellow arrow) in the nasal sector of the optic disc. **(E)** En face OCT displays a hyperreflective reniform mass encircling the optic disc. **(F)** OCTA shows flow signal within PHOMS. **(G)** B-ultrasound shows small high echoes without posterior sound shadow at the retinal level of the optic disc.

Here we summarize characteristic features of PHOMS on OCT (Figure 1).

#### 2.1.1 Location

PHOMS are located on the peripapillary region, usually partially or 360° around the optic disc (13, 18, 19). A hyporeflective outer nuclear layer separates PHOMS from the above inner retina. On the B-scan through the optic disc center, PHOMS are located on either or both sides of the BMO, while on a transverse or longitudinal B-scan across the margin of the optic disc, PHOMS are located above the continuous Bruch membrane (Figures 1C, D). PHOMS are mostly distributed on the nasal sector of the optic disc and are therefore more likely to be detected by nasal volume scanning of the optic nerve head (ONH) (13, 15, 20).

#### 2.1.2 Signal

PHOMS are manifested as diffuse, solid, and internally slightly uneven hyperreflectivity, which suggests that they have an inhomogeneous and complex internal structure. The reflectivity of PHOMS is similar to that of the retinal nerve fiber layer (RNFL) and ganglion cell layer (GCL), supporting the hypothesis that PHOMS may correspond to dilated axons laterally herniating into the peripapillary retina. Besides, it has been reported that 95.2% of PHOMS in children have small internal hyperreflective spots, which may be calcium within PHOMS (21).

#### 2.1.3 Shape

On the OCT B-scan, PHOMS are oval with smooth edges, separated from the surrounding retinal tissue by hyporeflective boundaries (Figures 1C, D). They appear as continuous structures in successive cross-sectional scans instead of dispersed masses (5). However, PHOMS vary in size in different cross sections, so the previous description of them as doughnuts may not be appropriate, which are standard toruses (18). On En face OCT, they appear as hyperreflective reniform masses or annuli encircling the optic disc (19).

#### 2.1.4 Effect on surrounding tissues

In the cross-section of the optic disc, PHOMS appear as masses with a certain “occupying” effect and often cause the overlying retinal tissue to shift outward and upward, thus forming a curved shape like a ski slope or boot (1, 22). However, the position of the Bruch membrane beneath PHOMS usually doesn't change significantly. Whether the presence of PHOMS is associated with peripapillary RNFL thickness is still under debate. Some studies have suggested that PHOMS are related to the thinning of the RNFL above them, while others have revealed that PHOMS don't affect the thickness of the RNFL or GCL (2, 9, 23, 24). Longer follow-up is essential to reveal whether PHOMS can cause RNFL atrophy.

#### 2.1.5 Differential diagnosis

**Figure 2:**
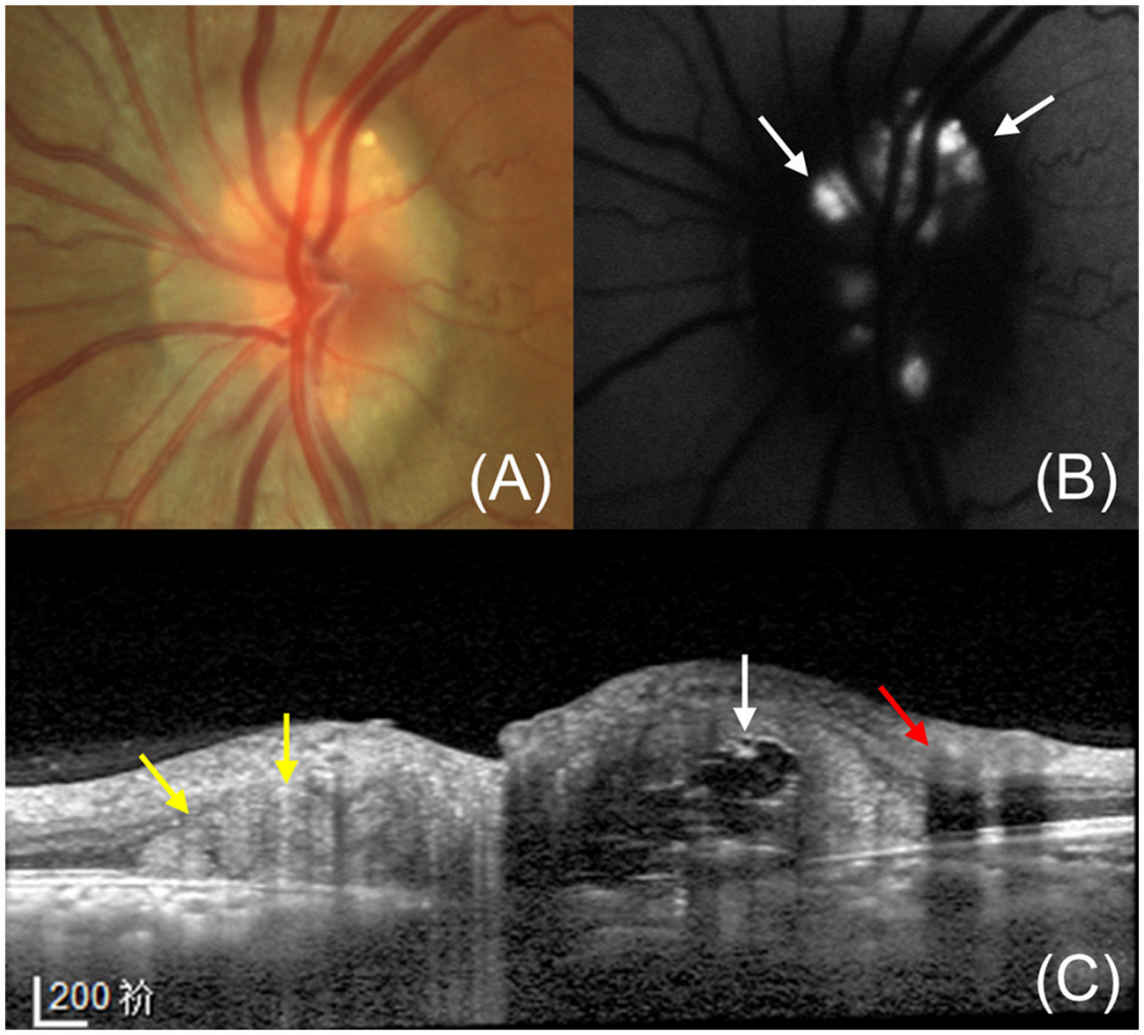
A 36-year-old female with PHOMS and ODD. **(A)** Fundus photography shows a shiny, irregular crystalline form on the optic disc with a blurry edge. **(B)** Fundus autofluorescence shows the typical hyperautofluorescence of the ODD at the disc. **(C)** Enhanced depth imaging OCT exhibits the ODD (white arrow), superficial optic disc vessels (red arrow) and accompanying PHOMS (yellow arrow). A typical ODD shows the hyperreflective shell and hyperreflective core while a large PHOMS is identifiable by its hyperreflective ovoid mass-like structure. The optic disc vessels show circular structures with underlying shadows.

Though PHOMS have typical characteristics on OCT, they should be distinguished from superficial optic disc vessels and ODD on the B-scan (Figure 2). Blood vessels present oval-shaped hyperreflective structures similar to PHOMS when imaged cross-sectionally, but they can be distinguished by their superficial position and underlying shadows (1, 5). In addition to optic disc vessels, ODD is also easily confused with PHOMS. It appears as an irregular structure with a hyporeflective interior circled by a hyperreflective margin (1, 5). Notably, ODD is always accompanied by the presence of PHOMS (20, 23).

### 2.2 Optical coherence tomography angiography

Optical coherence tomography angiography (OCTA) provides rapid and non-invasive visualization of retinal and choroidal vasculatures by detecting blood flow signals in the vascular lumen (25). Several studies have demonstrated the presence of complex vascular structures within PHOMS by OCTA (15, 26–28). It was speculated that the vascular complex may be related to a displacement of the deeper vessels in the optic disc into the retina or be secondary to neovascularization (26). In addition, the presence of PHOMS affects the microvascular structure of the optic disc region. Kim et al. (19) discovered that the vessel density of the radial peripapillary capillary above PHOMS showed a characteristic reduction in nasal C-shaped in a quarter of patients with PHOMS. Moreover, the size of PHOMS was negatively correlated with the vessel density of the radial peripapillary capillary (19). Quantitative analysis of the optic disc blood flow in children showed that the vessel density in the ONH was significantly reduced in the large PHOMS (height ≥ 500 μm) group (23). The squeezing caused by PHOMS or co-existing ODD or both may reduce or redistribute the blood flow in the optic disc area. The marked decline of the vessel density in optic discs suggests that the microvascular changes may be an early indicator of functional changes before changes in RNFL or GCL thickness (23).

### 2.3 Additional imaging techniques

Under fundoscopy, a C-shaped or O-shaped halo can be seen at the edge of the optic disc, especially on the nasal side (29). Infrared reflectance (IR) imaging exhibits a clear ring structure corresponding to the PHOMS edges (21). ODD Consortium and some other scholars believed that PHOMS were undetectable by ultrasound and autofluorescence (1, 28, 30). However, Mezad-Koursh et al. (21) found that PHOMS in children exhibited small high echoes without posterior sound shadow at the retinal level of the optic disc instead of the deep layer on B-ultrasound. The minimum gain for detecting PHOMS was 56 dB and lower gains were required to visualize larger PHOMS (21). In addition, they observed hyperautofluorescence points that corresponded to the hyperreflective spots inside PHOMS on OCT (21). Fluorescein angiography demonstrated high fluorescence in the peripapillary region without leakage, indicating fluorescein staining of bulging RNFL tissue (22, 28). Larger studies are needed to clarify the manifestation of PHOMS in multimodal imaging, especially in B-ultrasound and AF, which are important tools for the differential diagnosis of ODD.

## 3 Pathophysiology of PHOMS

Although there is a dearth of direct research on the histopathology of PHOMS, investigations into pathological sections of PHOMS-related ailments such as papilledema and ODD can serve as valuable tools in advancing our understanding of PHOMS. Histological studies of the ONH have demonstrated swollen and vacuolated optic nerve fibers curving and expanding above the BMO to form an ovoid mass-like structure before exiting the lamina cribrosa (5, 31, 32). This structure causes adjacent retinas to be displaced outwardly, upwardly or even folded (5). Significantly, the mass structure in the pathological sections is almost identical to the location and shape of PHOMS on OCT (33). Positive S100 immunostaining further confirmed that this structure consisted of the bulging nerve fibers (33). Both radioisotopes and electron microscopy showed that markers of stalled axoplasmic flow transport could be detected within distended axons, indicating that PHOMS may be the products of axoplasmic stasis (31, 34–37).

The pathologic manifestations are not identical in different diseases associated with PHOMS, which reveals different mechanisms of PHOMS formation. In ODD, acellular and calcified deposits are observed anterior to the lamina cribrosa, which may impede normal axoplasmic transport and result in the formation of PHOMS (32). In patients with myopia and TDS, the tilted optic disc causes a protruding Bruch membrane on the nasal side of the optic disc. Dramatic bending of the nerve fibers as they enter the lamina cribrosa can result in chronic tensile damage, leading to stagnant axoplasmic flow (5, 8). In papilledema, increased cerebrospinal fluid pressure pressurizes the optic nerve and stagnates the axoplasmic flow (31). Besides, it's observed that the dilated nerve fibers are surrounded by a large number of dilated small veins and capillaries, and exuded interstitial fluid exacerbates the compression and swelling of the nerve fibers (31). In addition. Rao (38) observed demyelination of optic nerve fibers and infiltration of vast inflammatory cells in pathologic sections of an ON model, which may lead to an acute axoplasmic flow stasis of the nerve fibers.

In brief, a variety of diseases that cause mechanical stress of optic nerve fibers or inflammatory response in ONH can all result in a stasis of the axoplasmic flow, which contributes to the formation of PHOMS.

## 4 PHOMS in healthy people

**Figure 3:**
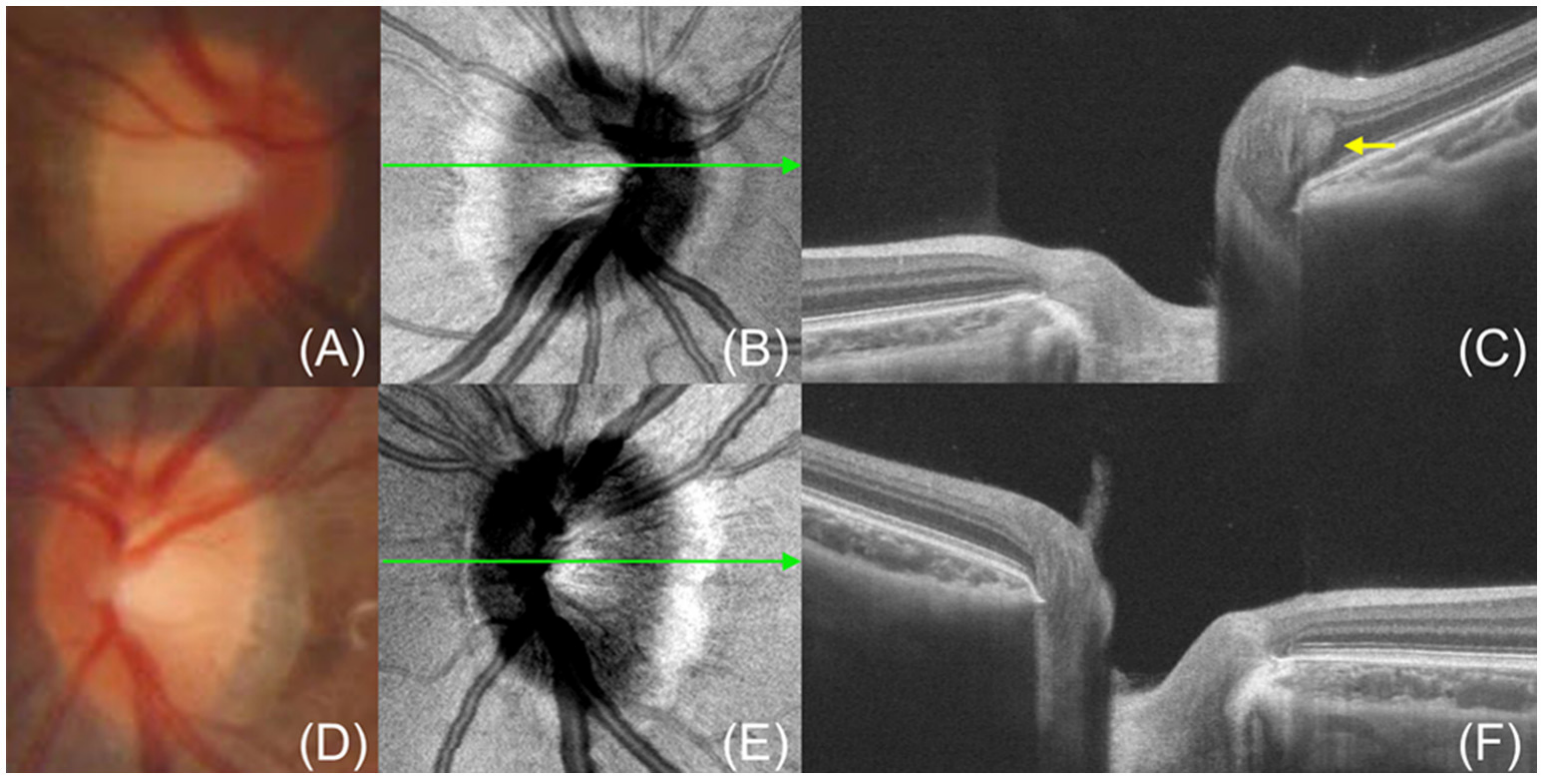
A 9-year-old girl without any ocular diseases has PHOMS in her right eye. **(A, D)** Fundus photography shows clear edges of the optic discs. **(B, E)** En face OCT shows a nasal “C”-shaped hyperreflective ring surrounding the optic disc in the right eye **(B)** but no hyperreflective ring in the left eye **(E)**. **(C, F)** OCT exhibits PHOMS (yellow arrow) in the nasal sector of the optic disc in the right eye **(C)** and no PHOMS in the left eye **(F)**. The green line indicates the OCT scan position.

Though PHOMS are believed to be secondary to axoplasmic stasis, it's unclear whether they are compensatory physiologic phenomena or pathological manifestations. Recent studies have revealed that PHOMS can appear in the optic discs of healthy humans (Figure 3). Gernert et al. (14) found that PHOMS were present in 4% of healthy people though Petzold et al. (39) reported no PHOMS in a healthy control group containing 59 subjects. In a cohort of 1,407 children aged 11–12 years without ODD or ODE, 8.9% had PHOMS in at least one eye (13). Several studies have revealed that PHOMS was the most common cause of pseudopapilloedema in children and adults (21, 40). Recently, Wang et al. (15) demonstrated that PHOMS appeared in 18.9% of normal subjects, mainly in both eyes, and the size of PHOMS was negatively correlated with the cup/disc ratio in normal participant groups. It should be noted that most eyes demonstrate normal morphology of optic discs despite the presence of PHOMS (15). The small size of PHOMS in healthy individuals is a possible explanation for this phenomenon. However, the definition of a healthy person in these studies only excludes ophthalmic disease history. Some anatomical abnormalities such as small optic discs and tilted discs were not taken into account, which may be important risk factors for the development of PHOMS. Future research about the correlation between PHOMS and optic disc morphological characteristics of healthy individuals may be beneficial for deepening our understanding of PHOMS. Besides, it remains to be studied whether the presence of PHOMS affects visual function of healthy people.

## 5 PHOMS and associated diseases

The existence of PHOMS in a variety of disease entities indicates that they are non-specific imaging characteristics rather than a specific disease (5). Recently, the disease spectrum reported PHOMS has been expanding. According to the location of the lesions, we divided diseases associated with PHOMS into optic disc heteroplasia, optic neuropathy, and central nervous system disease.

### 5.1 PHOMS associated with optic disc heteroplasia

#### 5.1.1 ODD

ODD is a congenital heteroplasia of optic discs involving one or both eyes, most often in small, crowded discs (41). The prevalence of ODD is about 2% in human pathological examination (32, 42). It is believed that the formation of ODD is the result of extracellular calcium deposition caused by abnormal axonal metabolism (32). PHOMS are different entities from ODD and they have different multimodal imaging features (Table 1). Notably, PHOMS showed a high incidence in patients with ODD. Teixeira et al. (20) reported that PHOMS were present in up to 90% of pediatric patients with ODD and they were not correlated with RNFL thickness. Moreover, the distribution of PHOMS coincided with ODD, predominantly on the nasal sector of the optic disc (20). In adults, the incidence of PHOMS in ODD is reported to range from 47 to 63% (10, 39). Jorgensen et al. (7) retrospectively analyzed 321 affected eyes of ODD patients of all ages and observed coexisting PHOMS in 77%. The high incidence rate of PHOMS in ODD may be due to their common anatomical basis of small optic discs and the identical pathological mechanism of axoplasmic transport stasis. In addition, the presence of ODD may have increased mechanical compression of the optic nerve fibers, resulting in aggravating axoplasm stasis. PHOMS appear more frequently in superficial ODDs than in buried ODDs, which may be because superficial ODDs are usually large and located in the area above and below the BMO, whereas buried ODDs usually involve smaller ODDs located beneath the BMO (7). It was reported that both the prevalence and size of PHOMS were negatively correlated with age in ODD patients (7). The potential mechanism may be that age-related axon loss reduces the compression of nerve fibers in the scleral canal, thereby improving axoplasmic stasis. Jorgensen et al. (7) didn't find a correlation between the volume of PHOMS and the size of BMO in ODD though it was reported that PHOMS were more likely to occur in children with small BMO. They speculated that some other anatomical characteristics of the optic disc could be involved in the pathophysiology of PHOMS. Further studies are needed to reveal the risk factors for PHOMS in ODD and whether they diminish or subside with age-related ONH depressurization.

**Table 1:** Summary of key imaging differences between PHOMS and ODD.

	**PHOMS**	**ODD**
Fundoscopy	A C-shaped or O-shaped peripapillary halo	Shiny, irregular crystalline forms within the optic disc
OCT	Partially or 360° around the optic disc, mostly nasally; Ovoid, mass-like structures; Diffuse and internally slightly uneven hyperreflectivity	A lobulated, intricate configuration or aggregation of ODD clusters within the optic nerve head; A hyporeflective interior circled by hyperreflective margins
OCTA	Dense microvascular network	No microvascular network
AF	Undetectable or small hyperautofluorescence points	Hyperautofluorescence structures
Ultrasound	Undetectable or small high echoes without posterior sound shadow at the retinal level of the optic disc	Typical high echoes accompanied by a posterior sound shadow deep within the optic disc

PHOMS, peripapillary hyperreflective ovoid mass-like structures; ODD, optic disc drusen; OCT, optical coherence tomography; OCTA, optical coherence tomography angiography; AF, autofluorescence.

#### 5.1.2 Optic disc tilt

Both congenital or acquired diseases can lead to optic disc tilt. TDS, characterized by tilting of the optic disc, is a congenital abnormality caused by delayed closure of the embryonic fissure (43). As early as 2014, Pichi et al. (8) reported that PHOMS were present in the nasal optic disc sector in 39.5% of children with TDS, although they were named “dome-shaped hyperreflective structures” at the time. They noted that nearly half of the PHOMS showed a reduction in height in the following year (8). PHOMS corresponded to the early abnormal visual field and 46.7% of affected eyes had persistent visual field defects after refractive correction, possibly due to damaged axoplasmic transport caused by prolonged bending of nerve fibers (8). In addition to TDS, an acquired tilted disc caused by scleral stretching is also a feature of myopia (44). Lyu et al. (45) reported for the first time that PHOMS were significantly associated with the degree of myopia and the magnitude of ONH tilt angle in children. Subsequently, Behrens et al. (13) reported that the prevalence of PHOMS was 8.9% in a children cohort of 11–12 years old. Similar to Lyu et al., they revealed that the presence and increase of ONH tilt, as well as the increase of myopia can significantly elevate the risk of PHOMS (13). A recent high myopia cohort study reported a 29.5% prevalence of PHOMS in non-pathological high myopia, and 13.69% of visual field defects corresponded to the location of PHOMS on OCT (46). Long-term follow-up of several cases of myopic children showed that PHOMS formed and developed as myopia deepens and optic disc tilt increases (45, 47). Therefore, the myopic shift may contribute to the formation of PHOMS by mediating optic disc tilt (13, 45). Optic nerve fibers bend sharply as they descend into the scleral canal because of the protruding Bruch's membrane in the tilted disc, and they are also subjected to nasal dragging of the laminar cribrosa and stretching of the temporal sclera (5). As a result, PHOMS appear as the axoplasmic flow of nerve fibers stagnates under mechanical stress. Moreover, it remains to be studied whether PHOMS impair the patients' visual function and whether the size of PHOMS changes as myopia deepens.

### 5.2 PHOMS associated with optic neuropathy

#### 5.2.1 NAION

NAION is an ischemic injury to the optic nerve due to impaired circulation in the posterior ciliary artery supplying the anterior part of the ONH (48). It is a multifactorial ischemic lesion, involving local anatomy factors and various systemic factors (49). NAION occurs mostly in small and crowded ONHs, which is more pronounced in younger patients (10). A retrospective study showed that PHOMS were found in five of nine patients with NAION and they were not associated with the thickness of RNFL and GCL (50). Hamann et al. (10) studied 65 NAION patients under the age of 50 and reported that PHOMS were present in 54% of NAION with ODD (ODD-NAION) patients and in 28% of NAION patients without ODD (nODD-NAION). Similarly, Johannesen et al. (51) indicated that ODD-NAION patients had a higher incidence of PHOMS compared to nODD-NAION patients, which may be attributed to increased mechanical compression of the optic nerve fibers caused by small ONHs and ODD. Recently, Wang et al. (15) demonstrated firstly that the prevalence of PHOMS was significantly higher in acute nODD-NAION eyes (43.48%) and fellow eyes (28.20%) than in normal eyes (11.76%). The majority of PHOMS disappeared within the next 1–2 months in patients with acute NAION, while maintained stability in the fellow eyes (15). PHOMS may be connected with the severity of optic disc edema (ODE) and become smaller or disappear as ODE subsides (15). Notably, the high prevalence of PHOMS in unaffected fellow eyes of patients with acute NAION may be associated with small optic discs (15). It is not clear whether the structure PHOMS is a novel independent risk factor for NAION or a secondary product of ODE in the acute stage. In addition, we look forward to further studies to reveal whether PHOMS affect visual function prognosis in NAION patients.

#### 5.2.2 LHON

LHON is a type of mitochondrial hereditary disease characterized by degenerative changes following pathological damage to retinal ganglion cells and their axons (52). Acute LHON is characterized by RNFL swelling, which may be connected to axoplasmic stagnation and increased mitochondrial biogenesis (53). Recently, Borrelli et al. (12) first described the presence of PHOMS in 12 of 21 (57.1%) eyes with LHON. All PHOMS were distributed on the temporal sector of the optic disc, possibly because that the small size of temporal optic nerve fibers makes them more vulnerable to mitochondrial malfunction (12). Besides, PHOMS were significantly associated with RNFL thickening and resolved within 12 months in most cases along with edema disappeared, which indicated that ODE played a crucial role in PHOMS formation (12). It remains to be studied whether mitochondrial dysfunction is involved in the formation of PHOMS.

#### 5.2.3 Optic nerve tumor

Optic nerve tumors are a rare group of optic nerve diseases, including optic nerve sheath meningioma and optic nerve glioma, optic disc tumors, optic schwannomas, and infiltrating optic neuropathy caused by metastasis or infiltration of malignant tumors, among which optic nerve sheath meningioma and optic nerve glioma are more common (54). In 2015, Lee et al. (55) reported three cases of optic nerve tumors combined with ODD, including optic disc melanocytic tumor, optic nerve meningioma, and optic nerve glioma. It now appears that the ODD they refer to is actually PHOMS. Heath Jeffery et al. (22) also presented a patient with optic nerve sheath meningioma and PHOMS, and the existence of PHOMS did not appear to impair the patient's vision during the observation for up to 20 years. It is speculated that tumors involving the optic nerve can lead to compression of nerve fibers, axoplasmic flow stasis, and subsequent development of PHOMS. However, larger studies are necessary to determine the exact relationship between PHOMS and optic nerve tumors.

#### 5.2.4 Diabetic papillopathy

Diabetes mellitus can cause many vision-threatening ocular complications, such as proliferative diabetic retinopathy and macular edema (56). Diabetic papillopathy is another potential complication characterized by unilateral or bilateral ODE due to vessel leakage and axon swelling (57). Becker et al. reported that one patient with diabetes mellitus developed severe acute bilateral ODE shortly after initiation of intensive glucose-lowering therapy (58). PHOMS were observed in both eyes accompanied by ODD at the initial visit, and they were present with lower height at a follow-up 9 months later (58). Heath Jeffery et al. (22) noted PHOMS in a male with presumed diabetic papillopathy and it partially involuted after 12 months. However, it is unclear whether PHOMS appeared before or after diabetic papillopathy. The reduction of PHOMS after ODE receding may indicate that they are secondary products of ODE.

### 5.3 PHOMS associated with central nervous system diseases

#### 5.3.1 Intracranial hypertension

Intracranial hypertension is a common clinical syndrome in which intracranial pressure (ICP) exceeds the compensatory range, including idiopathic intracranial hypertension (IIH) and secondary intracranial hypertension caused by central nervous system disorders such as brain tumors, hydrocephalus, infection, trauma, hemorrhage, or venous thrombosis (59). It is characterized by headache, vomiting, and papilledema. Of all the diseases associated with PHOMS that have been reported, PHOMS occurs most frequently in IIH (9). Petzold et al. (39) observed that PHOMS were present in eight of 13 patients with intracranial hypertension. A retrospective study incorporating 32 patients with IIH revealed that 81.3% of IIH patients had PHOMS (9). The potential mechanism could be that elevated cerebrospinal fluid pressure reverses the trans-lamina cribrosa pressure difference, resulting in a stagnant axoplasmic flow. Regression of PHOMS was not observed, probably because of lacking OCT examinations in the acute phase and within 3 months (9). Differently, Fraser et al. (5) and Malmqvist et al. (60) noted that PHOMS diminished or even disappeared in IIH patients after weight loss and acetazolamide treatment. In addition, there were no significant differences in RNFL thickness and GCL volume between patients with or without PHOMS, indicating that PHOMS did not cause pathological damage to the optic disc and macular nerve fibers (9). Bassi et al. (61) retrospectively analyzed 23 patients with bilateral papilledema secondary to elevated ICP, including 20 patients with IIH, two patients with obstructive hydrocephalus, and 1 patient with communicating hydrocephalus, and observed the presence of PHOMS in 21 eyes (45.65%). PHOMS were most commonly observed in the eyes of patients with ICP in the 251–350 mmH2O range (61). The lower incidence of PHOMS than previously reported may be because PHOMS cannot be detected due to the shadow of thickened RNFL in acute IIH cases with significant papilledema. Further research is essential to evaluate whether the magnitude of PHOMS can reflect the level of intracranial pressure non-invasively. Prospective studies with larger sample sizes may reveal alternations in PHOMS as papilledema subsides and whether PHOMS contribute to visual field defects in papilledema patients.

#### 5.3.2 Inflammatory demyelinating disease

Multiple sclerosis (MS) is an autoimmune disease characterized by inflammatory demyelinating lesions of the white matter of the central nervous system (62). It most commonly involves the periventricular white matter, optic nerves, spinal cord, brainstem, and cerebellum. A prospective longitudinal study including 212 patients with MS, 59 healthy controls, and 267 patients without MS indicated that the incidence of PHOMS was significantly higher in MS patients (16%) than in healthy controls (0%) (39). At 2 years of follow-up, most PHOMS remained stable while a few PHOMS increased in size or started to appear (39). PHOMS didn't affect the thickness of RNFL and macular GCL in MS patients (39). Wicklein et al. (63) revealed that PHOMS presented in 18.3% of patients with early relapsing-remitting MS and 19.7% of primary progressive MS in a cross-sectional study. PHOMS were associated with disease duration and progression of disability in PPMS (63). However, the presence of PHOMS is not associated with a history of ON in MS (39, 63). In addition to MS, PHOMS also appears in two other demyelinating diseases. Gemert et al. (14) detected PHOMS in 17% of AQP4-IgG-positive neuromyelitis optica spectrum disease patients and 14% of MOG-IgG-associated disease patients, comparable to the prevalence of PHOMS in MS patients. They revealed that the development of PHOMS was not associated with disease duration, disability, or optic nerve axonal degeneration (14). Recently, Aziria et al. (11) retrospectively analyzed 115 eyes with ON involving multiple different etiologies and reported that PHOMS is not a common structure in ON patients, which support a non-inflammatory mechanism in the formation of PHOMS (11). Therefore, the formation mechanism of PHOMS in inflammatory demyelinating lesions may involve stasis of axoplasm flow in optic nerve fibers, impaired circulation in the lymphatic system, and increased pressure gradient across the laminar cribrosa (14, 39, 63). Further studies are requisite to evaluate if PHOMS correlates with the severity and prognosis of central nervous system demyelinating diseases.

### 5.4 Other associated retinal and orbital diseases

In addition to optic nerve diseases and central nervous system diseases mentioned above, PHOMS can be seen in some other retinal and orbital diseases causing compartment syndrome or optic nerve compression. Dai et al. (50) revealed that PHOMS were present in 13% of patients with retinal vascular obstruction. However, they didn't report whether a masculine symptom of ODE was a necessary prerequisite for the development of PHOMS (50). Moreover, Xie et al. (28) observed PHOMS in two eyes with white dot syndrome and one eye with macular neovascularization. It is uncertain whether PHOMS are present before or after the onset of the diseases. Though it has not yet been reported, PHOMS may be present in other diseases that can cause ODE, such as retinal vasculitis, hypertensive retinopathy, posterior uveitis, posterior scleritis, thyroid-associated ophthalmopathy, orbital inflammation, tumors, etc. More research is needed to determine the broad disease spectrum of PHOMS and whether their presence affects patients' visual prognosis.

## 6 Conclusion and prospect

For a long time, PHOMS were considered a subtype of ODD (2–4). However, both multimodal imaging and pathologic studies have shown PHOMS to be distinct from ODD. PHOMS appear in a wide spectrum of diseases, including optic disc heteroplasia, various optic neuropathy, and central nervous system diseases. It remains to be further explored whether PHOMS exist in other retinal diseases and orbital diseases that cause ONH compartment syndrome or optic nerve compression. In addition to the diseases mentioned above, PHOMS have also been reported in healthy humans (13–15). There is no doubt that PHOMS are markers of axoplasmic stasis in the ONH. However, it's not certain whether the presence of PHOMS is just a compensatory physiological phenomenon or a pathological change affecting visual function. Importantly, though PHOMS show a high incidence in pseudopapilloedema, they should not be used to distinguish pseudopapilloedema from ODE and papilledema in clinical practice, because PHOMS can occur in all three situations. Further studies are required to explore whether the presence of PHOMS can reflect the severity of diseases and indicate the patients' visual prognosis.

## Author contributions

DX: Conceptualization, Visualization, Writing – original draft. TL: Writing – review & editing. YM: Writing – review & editing. YX: Supervision, Writing – review & editing. CC: Supervision, Writing – review & editing.
